# Prevalence of SARS-CoV-2 Infection in Children by Antibody Detection in Saliva: Protocol for a Prospective Longitudinal Study (Coro-Buddy)

**DOI:** 10.2196/27739

**Published:** 2021-10-08

**Authors:** Yudi T Pinilla, Evelyn Friessinger, Johanna Marie Griesbaum, Lilith Berner, Constanze Heinzel, Käthe Elsner, Rolf Fendel, Jana Held, Andrea Kreidenweiss

**Affiliations:** 1 Institut für Tropenmedizin Universitätsklinikum Tübingen Tübingen Germany; 2 Centre de Recherches Médicales de Lambaréné Lambaréné Gabon; 3 German Center for Infection Research, partner site Tübingen Tübingen Germany

**Keywords:** SARS-CoV-2, COVID-19, antibody, saliva, children, epidemiology

## Abstract

**Background:**

The world has been confronted with the COVID-19 pandemic for more than one year. Severe disease is more often found among elderly people, whereas most young children and adolescents show mild symptoms or even remain asymptomatic, so that infection might be undiagnosed. Therefore, only limited epidemiological data on SARS-CoV-2 infection in children and young adults are available.

**Objective:**

This study aims to determine the prevalence of SARS-CoV-2 antibodies in children from the city of Tübingen, Germany, and to measure the incidence of new cases over 12 months.

**Methods:**

SARS-CoV-2 antibodies will be measured in saliva as a surrogate for a previous SARS-CoV-2 infection. Children will be sampled at their preschools, primary schools, and secondary schools at three time points: July 2020, October to December 2020, and April to July 2021. An adult cohort will be sampled at the same time points (ie, adult comparator group). The saliva-based SARS-CoV-2–antibody enzyme-linked immunosorbent assay will be validated using blood and saliva samples from adults with confirmed previous SARS-CoV-2 infections (ie, adult validation group).

**Results:**

The first study participant was enrolled in July 2020, and recruitment and enrollment continued until July 2021. We have recruited and enrolled 1850 children, 560 adults for the comparator group, and 83 adults for the validation group. We have collected samples from the children and the adults for the comparator group at the three time points. We followed up with participants in the adult validation group every 2 months and, as of the writing of this paper, we were at time point 7. We will conduct data analysis after the data collection period.

**Conclusions:**

Infection rates in children are commonly underreported due to a lack of polymerase chain reaction testing. This study will report on the prevalence of SARS-CoV-2 infections in infants, school children, and adolescents as well as the incidence change over 12 months in the city of Tübingen, Germany. The saliva sampling approach for SARS-CoV-2–antibody measurement allows for a unique, representative, population-based sample collection process.

**Trial Registration:**

ClinicalTrials.gov NCT04581889; https://clinicaltrials.gov/ct2/show/NCT04581889

**International Registered Report Identifier (IRRID):**

DERR1-10.2196/27739

## Introduction

The end of 2019 was marked by the emergence of a novel betacoronavirus, called SARS-CoV-2, which caused an outbreak in the Chinese city of Wuhan [1] and eventually spread to more than 180 countries. On March 11, 2020, the World Health Organization declared the COVID-19 outbreak a global pandemic [2,3]. The novel virus spreads rapidly by efficient human-to-human airborne transmission [4]. Symptoms mainly include fever, cough, myalgia, loss of smell or taste, and a severe manifestation of pneumonia, but other symptoms of respiratory tract infections can also occur [5-7]. Children infected with SARS-CoV-2 typically experience mild disease or are asymptomatic [8]; few pediatric cases of severe or fatal COVID-19 have been reported [7]. In Germany, a total of 3,756,497 laboratory-confirmed SARS-CoV-2 infections, including 117,482 deaths, have been recorded as of July 2021 by the Robert Koch Institute in Berlin, Germany [9]. Among those, 225,270 were children under 10 years of age (6.0%) and 366,472 (9.8%) were adolescents aged 10 to 19 years [9]. At the time, the diagnostic strategy focused primarily on testing symptomatic individuals, and children may have been underrepresented in such records. Also, hospital-based studies or case series are biased toward recruitment of a selected group, and the fraction of identified children infected with SARS-CoV-2 is not generalizable to the larger population [10].

Epidemiologic surveys require an unbiased, sufficiently large, representative sampling approach. Screening for SARS-CoV-2–reactive antibodies allows for the retrospective identification of virus exposure and is amenable to large cohorts [11]. However, the prerequisite of blood sampling [12] is a hurdle to the screening of large pediatric cohorts, particularly outside of the medical context. Interestingly, antibodies are also secreted by mucosal tissues, and SARS-CoV-2–specific antibodies can be detected in saliva and other body fluids [13]. Saliva sampling as a noninvasive method (ie, it does not cause disturbance) for SARS-CoV-2 antibody measurements and is an elegant approach to rapidly assess the prevalence of SARS-CoV-2 infection in vulnerable cohorts at numbers appropriate for epidemiologic investigations [14]. Studies have reported the use of saliva not only for detection of respiratory viruses, including coronaviruses and SARS-CoV-2 [15], but also for detection of antibodies against measles, rubella, mumps, and hepatitis [16-18]. The number of seroprevalence studies benefiting from noninvasive saliva sampling is increasing [19].

This study assesses the prevalence of SARS-CoV-2 antibodies as a surrogate for previous infections in children (aged 1-18 years) and measures the change of incidence over a 12-month period in a defined study area. The study takes place in Tübingen, a middle-sized university city in the Federal State Baden-Württemberg, Germany. Saliva samples from children were collected three times in preschools, primary schools, and secondary schools: summer 2020, 6 months later, and 12 months later (ie, before and after winter 2020-2021). SARS-CoV-2 antibodies will be measured in-house using a pre-established enzyme-linked immunosorbent assay (ELISA).

## Methods

### Study Design

**Figure 1:**
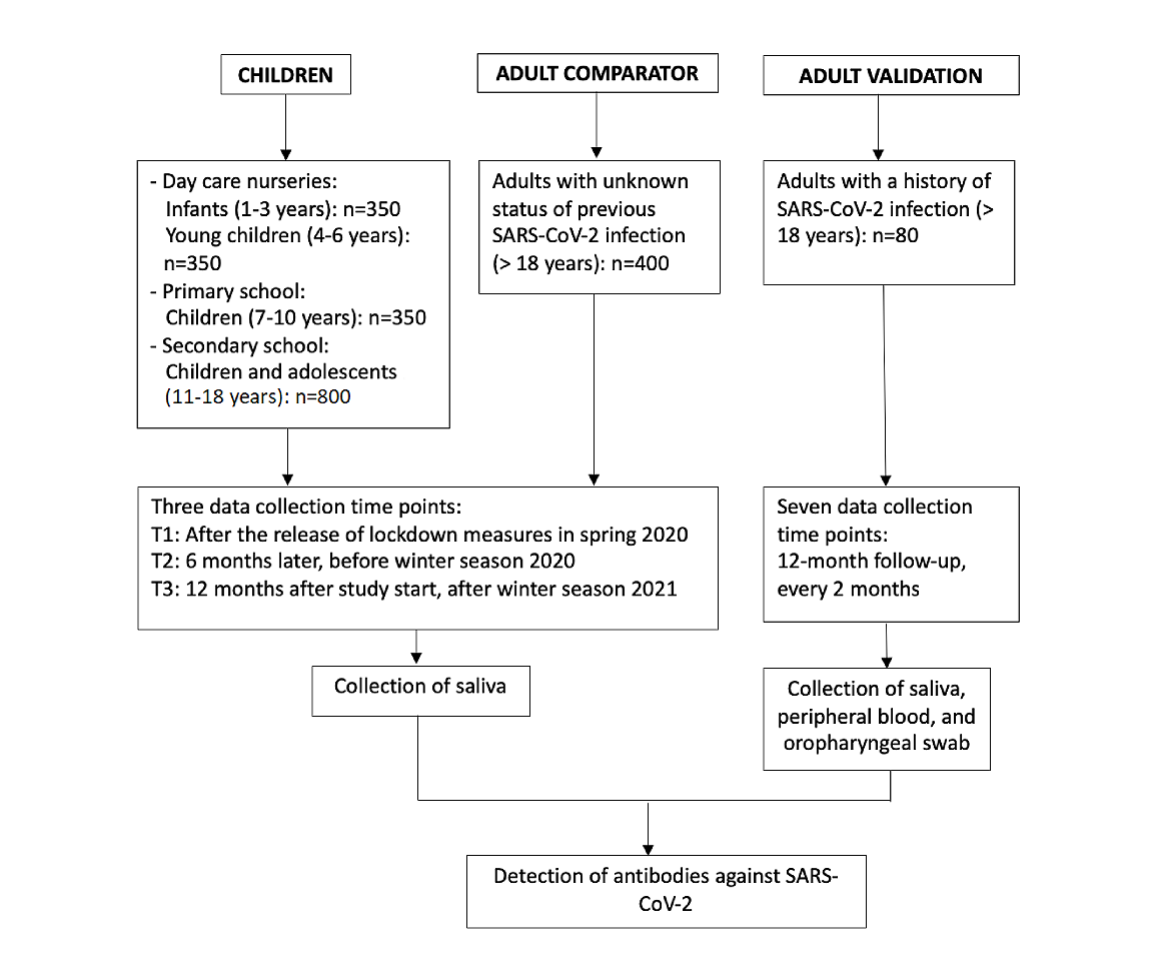
Study flowchart for participant sampling. T: time point.

Our study, titled Coro-Buddy (Coronavirus and Antibody Buddy), is a longitudinal, prospective, observational study to determine the prevalence of SARS-CoV-2 antibodies as a surrogate for previous infections in children and adolescents, and the change of antibody incidence over 12 months. Saliva samples from each study participant were collected at three time points: time point 1 (T1), after the release of lockdown measures in spring 2020 (ie, summer 2020); time point 2 (T2), before the winter season 2020; and time point 3 (T3), after the winter season 2021. An adult cohort (ie, adult comparator group) was sampled at the same time points to describe the seroepidemiological profile in adults. In addition, to establish and validate the SARS-CoV-2 antibody measurement process in saliva, an additional adult cohort group (ie, adult validation group) will have saliva and peripheral blood samples collected every 2 months at seven time points, over a 1-year follow-up. See Figure 1 for an overview of the study flow.

### Study Population

Children and adolescents were enrolled and sampled via preschools, primary schools, and secondary schools randomly spread over the Tübingen city area. In total, 1850 children and adolescents were enrolled: (1) preschools—infants aged 1 to 3 years (n=350) and young children aged 4 to 6 years (n=350), (1) primary schools—children aged 7 to 10 years (n=350), and (3) secondary schools—children and adolescents aged 11 to 18 years (n=800). Study participants and their families were informed about the study and its aims via their institutions. In addition to all relevant documents, families were also provided with a link to a video that explains the study (Multimedia Appendix 1) and procedures in an easy-to-understand way to ensure broad participation. Saliva was sampled after written informed consent has been given by parents or legal representatives. Assent from children aged 12 years and older was collected on the day of first sampling. Inclusion criteria include the following: aged 1 to 18 years, enrolled in an educational institution within the city of Tübingen, and written informed consent given or written assent given if the child is 12 years or older. Exclusion criteria include not attending a preschool or school in the city of Tübingen.

For the adult comparator group (n=400), any person aged 18 years and older living or working in Tübingen who had given written informed consent could be enrolled. For the adult validation group (n=40), the following individuals can participate in the study: those with a previous SARS-CoV-2 infection, confirmed by either quantitative polymerase chain reaction or by ELISA with a SARS-CoV-2–specific antibody at any time period before enrollment, who have given written informed consent. Blood and saliva samples are being collected every 2 months for 12 months (Figure 1).

### Sample Size

Tübingen has a total of 91,656 inhabitants; among them, 4910 are children under the age of 6 years and 8366 are children between the ages of 6 and 18 years (≤6 years: n=4061; 7-18 years: n=6621, as of December 31, 2019). Therefore, in total, 13,276 children were living in Tübingen at the time of the study; of these, we aimed to recruit 1850 children and young adults. In addition, 400 adults will be included as an adult comparator group. At the time of planning, 567 out of 100,000 inhabitants—as determined by the Robert Koch Institute in Berlin, Germany, on June 1, 2020—have had a confirmed SARS-CoV-2 infection in the county of Tübingen, but the number of unreported and asymptomatic infections is believed to be higher. Therefore, we expect to have at least 2.2 positive cases in the adult group; in the event that children are infected at an equal frequency, we expect to find at least 10.4 positive cases in children at T1.

### Questionnaire

A structured questionnaire with six questions addressing exposure to SARS-CoV-2 infection in the family was administered at each of the three time points to the parents and representatives of the children as well as to the participants in the adult comparator group (Multimedia Appendix 2).

### Saliva and Blood Collection

Saliva samples were collected from children 6 years and below using an ORACOL S14 saliva collection device (Malvern Medical Developments) by gently brushing the gumline for 2 minutes. For children 7 years and above, 3 mL of saliva was collected by spitting into a plastic tube; 30-mL multipurpose containers (item No. 201150; Greiner Bio-One) were used for this purpose. Saliva samples were kept on ice for a maximum of 3 hours before further processing. The ORACOL S14 saliva collection device was centrifuged at 2500 rpm for 10 minutes and the 30-mL multipurpose containers were centrifuged for 6 minutes. Supernatant was transferred into a 2-mL microtube and inactivated using a solvent and detergent treatment to a final concentration of 0.3% tri-*n*-butyl phosphate and 1% Triton X-100. Samples were immediately stored at –20 °C. Blood was collected by vein puncture into 9-mL lithium-heparin Monovettes; the plasma was obtained by centrifugation at 1400 rpm for 10 minutes and stored at –20 °C.

### Laboratory Analysis

Saliva and blood samples were processed according to standard procedures and stored at –20 °C until analysis by ELISA to identify SARS-CoV-2–reactive antibodies. A previously established and validated in-house ELISA to detect antibodies against SARS-CoV-2 in saliva was performed [20]. Blood samples from the validation group cohort will be analyzed using a certified commercial ELISA by EUROIMMUN to quantify immunoglobulin G reactive to the S1 domain of the of SARS-CoV-2 spike protein.

### Data Management

The case report form is the source document for all personal data. Each participant included in the study received a unique identifier. All data were collected on paper forms and were entered and pseudonymized into an electronic database.

### Data Analyses

Data will be typed into Excel (version 16.51; Microsoft), and graphics will be generated with Prism (version 9.1; GraphPad) and RStudio (version 1.2.5001) running R (version 4.0.4; The R Foundation). Simple descriptive statistical analyses will be conducted. Depending on the classification of the data, parametric or nonparametric tests will be used. Correlation and regression models will be analyzed using RStudio. The estimated prevalence will be computed and adjusted using the R package epiR (version 0.9-43).

### Ethics Approval and Consent to Participate

This study was conducted with the approval from, and consent form signed by, the parents and participants, according to the protocol that was reviewed and accepted by the Ethics Committee of the University Hospital Tübingen on June 24, 2020 (reference No. 20-231/BO1). Each study participant was protected against invasion of privacy. Written informed consent was obtained from the participants, with parental consent and participant assent from those 12 years of age and older. The trial was retrospectively registered at ClinicalTrials.gov (NCT04581889) on October 10, 2020.

### Data Availability Statement

Data will be available upon the study’s completion.

## Results

The first study participant was enrolled in July 2020, and recruitment and enrollment continued until July 2021. We have recruited and enrolled 1850 children, 560 adults for the comparator group, and 83 adults for the validation group. We have collected samples from the children and the adults for the comparator group at the three time points (T1-T3). We followed up with participants in the adult validation group every 2 months and, as of the writing of this paper, we were at time point 7. We will conduct data analysis after the data collection period.

An amendment to the protocol was submitted to, and approved by, the Ethics Committee of the Tübingen University Hospital for blood collection from antibody-positive children during data collection at T3. This study is expected to conclude in November 2021.

## Discussion

The world was confronted with the COVID-19 pandemic, which has become the biggest public health crisis of recent times, has caused a large number of deaths, and has become a burden on intensive care facilities [2]. The majority of affected persons are older than 18 years, although adolescents and children can also become infected, with recent estimates in the range of 5% [21-23]. Different study designs can provide information on the transmission dynamics in children. Large population-wide serosurveillance studies have been performed in Europe using blood samples [11,24,25]; however, in order to facilitate large population studies in school-aged children, especially if multiple samples are collected from within the same population, saliva samples should be considered. With these factors in mind, the Coro-Buddy study was developed as a prospective, longitudinal study and will report the prevalence of SARS-CoV-2 infection in children in a sample that is representative of the study area, Tübingen, a university city in the south of Germany. The study will also be able to report on the prevalence of previous SARS-CoV-2 exposure in the different age cohorts—1 to 6 years, 7 to 10 years, and 11 to 18 years—as well as the incidence of new cases at three different time points, representing the end of school closures in Germany in summer 2020, and before and after the winter 2020-2021 period.

Children were enrolled and sampled at their respective preschools or school centers to ensure representativeness of the young population in the study area. The infection rate will be determined by measuring antibodies against SARS-CoV-2 in saliva via noninvasive sampling. This approach is particularly amenable for community studies where the significance of the outcome increases by unbiased sampling. The study will also report on a validated saliva-based SARS-CoV-2–antibody ELISA procedure that can be used in future antibody prevalence studies. In addition, the study will report on the number of SARS-CoV-2 infections per institution; this data could contribute to comparative analysis with data collected beyond this study to better understand the pandemic retrospectively.

## Appendix

Multimedia Appendix 1 Coro-Buddy (Coronavirus and Antibody Buddy) study video.

Multimedia Appendix 2 Study questionnaire.

## Abbreviations

Coro-Buddy: Coronavirus and Antibody Buddy
ELISA: enzyme-linked immunosorbent assay
T1: time point 1
T2: time point 2
T3: time point 3

## References

[1] Yang X, Yu Y, Xu J, Shu H, Xia J, Liu H, Wu Y, Zhang L, Yu Z, Fang M, Yu T, Wang Y, Pan S, Zou X, Yuan S, Shang Y (2020). Clinical course and outcomes of critically ill patients with SARS-CoV-2 pneumonia in Wuhan, China: A single-centered, retrospective, observational study. Lancet Respir Med.

[2] (2020). WHO Director-General's opening remarks at the media briefing on COVID-19. World Health Organization.

[3] Amanat F, Stadlbauer D, Strohmeier S, Nguyen T, Chromikova V, McMahon M, Jiang K, Arunkumar GA, Jurczyszak D, Polanco J, Bermudez-Gonzalez M, Kleiner G, Aydillo T, Miorin L, Fierer DS, Lugo LA, Kojic EM, Stoever J, Liu STH, Cunningham-Rundles C, Felgner PL, Moran T, García-Sastre A, Caplivski D, Cheng AC, Kedzierska K, Vapalahti O, Hepojoki JM, Simon V, Krammer F (2020). A serological assay to detect SARS-CoV-2 seroconversion in humans. Nat Med.

[4] Chan JF, Yuan S, Kok K, To KK, Chu H, Yang J, Xing F, Liu J, Yip CC, Poon RW, Tsoi H, Lo SK, Chan K, Poon VK, Chan W, Ip JD, Cai J, Cheng VC, Chen H, Hui CK, Yuen K (2020). A familial cluster of pneumonia associated with the 2019 novel coronavirus indicating person-to-person transmission: A study of a family cluster. Lancet.

[5] Ciuca IM (2020). COVID-19 in children: An ample review. Risk Manag Healthc Policy.

[6] de Souza TH, Nadal JA, Nogueira RJN, Pereira RM, Brandão MB (2020). Clinical manifestations of children with COVID-19: A systematic review. Pediatr Pulmonol.

[7] Lai C, Liu YH, Wang C, Wang Y, Hsueh S, Yen M, Ko W, Hsueh P (2020). Asymptomatic carrier state, acute respiratory disease, and pneumonia due to severe acute respiratory syndrome coronavirus 2 (SARS-CoV-2): Facts and myths. J Microbiol Immunol Infect.

[8] Hong H, Wang Y, Chung H, Chen C (2020). Clinical characteristics of novel coronavirus disease 2019 (COVID-19) in newborns, infants and children. Pediatr Neonatol.

[9] COVID-19 (coronavirus SARS-CoV-2). Robert Koch Institute.

[10] Bailey LC, Razzaghi H, Burrows EK, Bunnell HT, Camacho PEF, Christakis DA, Eckrich D, Kitzmiller M, Lin SM, Magnusen BC, Newland J, Pajor NM, Ranade D, Rao S, Sofela O, Zahner J, Bruno C, Forrest CB (2021). Assessment of 135 794 pediatric patients tested for severe acute respiratory syndrome coronavirus 2 across the United States. JAMA Pediatr.

[11] Pollán M, Pérez-Gómez B, Pastor-Barriuso R, Oteo J, Hernán MA, Pérez-Olmeda M, Sanmartín JL, Fernández-García A, Cruz I, Fernández de Larrea N, Molina M, Rodríguez-Cabrera F, Martín M, Merino-Amador P, León Paniagua J, Muñoz-Montalvo JF, Blanco F, Yotti R, ENE-COVID Study Group (2020). Prevalence of SARS-CoV-2 in Spain (ENE-COVID): A nationwide, population-based seroepidemiological study. Lancet.

[12] Perera RA, Mok CK, Tsang OT, Lv H, Ko RL, Wu NC, Yuan M, Leung WS, Chan JM, Chik TS, Choi CY, Leung K, Chan KH, Chan KC, Li K, Wu JT, Wilson IA, Monto AS, Poon LL, Peiris M (2020). Serological assays for severe acute respiratory syndrome coronavirus 2 (SARS-CoV-2), March 2020. Euro Surveill.

[13] Isho B, Abe KT, Zuo M, Jamal AJ, Rathod B, Wang JH, Li Z, Chao G, Rojas OL, Bang YM, Pu A, Christie-Holmes N, Gervais C, Ceccarelli D, Samavarchi-Tehrani P, Guvenc F, Budylowski P, Li A, Paterson A, Yue FY, Marin LM, Caldwell L, Wrana JL, Colwill K, Sicheri F, Mubareka S, Gray-Owen SD, Drews SJ, Siqueira WL, Barrios-Rodiles M, Ostrowski M, Rini JM, Durocher Y, McGeer AJ, Gommerman JL, Gingras AC (2020). Persistence of serum and saliva antibody responses to SARS-CoV-2 spike antigens in COVID-19 patients. Sci Immunol.

[14] Pisanic N, Randad PR, Kruczynski K, Manabe YC, Thomas DL, Pekosz A, Klein SL, Betenbaugh MJ, Clarke WA, Laeyendecker O, Caturegli PP, Larman HB, Detrick B, Fairley JK, Sherman AC, Rouphael N, Edupuganti S, Granger DA, Granger SW, Collins MH, Heaney CD (2020). COVID-19 serology at population scale: SARS-CoV-2-specific antibody responses in saliva. J Clin Microbiol.

[15] Roque M, Proudfoot K, Mathys V, Yu S, Krieger N, Gernon T, Gokli K, Hamilton S, Cook C, Fong Y (2021). A review of nasopharyngeal swab and saliva tests for SARS-CoV-2 infection: Disease timelines, relative sensitivities, and test optimization. J Surg Oncol.

[16] Miller CS, Berger JR, Mootoor Y, Avdiushko SA, Zhu H, Kryscio RJ (2006). High prevalence of multiple human herpesviruses in saliva from human immunodeficiency virus-infected persons in the era of highly active antiretroviral therapy. J Clin Microbiol.

[17] Hettegger P, Huber J, Paßecker K, Soldo R, Kegler U, Nöhammer C, Weinhäusel A (2019). High similarity of IgG antibody profiles in blood and saliva opens opportunities for saliva based serology. PLoS One.

[18] Medina Cruz H, Salete de Paula V, Ferreira da Silva E, Rodrigues do Ó KM, Pádua Milagres FA, Santos Cruz M, Bastos FI, Corrêa da Mota J, Pollo-Flores P, Leal E, Coimbra Motta-Castro AR, Lewis-Ximenez LL, Lampe E, Melo Villar L (2019). Utility of oral fluid samples for hepatitis B antibody detection in real life conditions. BMC Infect Dis.

[19] Sullivan PS, Sailey C, Guest JL, Guarner J, Kelley C, Siegler AJ, Valentine-Graves M, Gravens L, Del Rio C, Sanchez TH (2020). Detection of SARS-CoV-2 RNA and antibodies in diverse samples: Protocol to validate the sufficiency of provider-observed, home-collected blood, saliva, and oropharyngeal samples. JMIR Public Health Surveill.

[20] Heinzel C, Pinilla YT, Elsner K, Friessinger E, Mordmüller B, Kremsner PG, Held J, Fendel R, Kreidenweiss A (2021). Non-invasive antibody assessment in saliva to determine SARS-CoV-2 exposure in young children. Front Immunol.

[21] Dong Y, Mo X, Hu Y, Qi X, Jiang F, Jiang Z, Tong S (2020). Epidemiology of COVID-19 among children in China. Pediatrics.

[22] Götzinger F, Santiago-García B, Noguera-Julián A, Lanaspa M, Lancella L, Calò Carducci FI, Gabrovska N, Velizarova S, Prunk P, Osterman V, Krivec U, Lo Vecchio A, Shingadia D, Soriano-Arandes A, Melendo S, Lanari M, Pierantoni L, Wagner N, L'Huillier AG, Heininger U, Ritz N, Bandi S, Krajcar N, Roglić S, Santos M, Christiaens C, Creuven M, Buonsenso D, Welch SB, Bogyi M, Brinkmann F, Tebruegge M, ptbnet COVID-19 Study Group (2020). COVID-19 in children and adolescents in Europe: A multinational, multicentre cohort study. Lancet Child Adolesc Health.

[23] (2021). COVID-19 in Children and the Role of School Settings in Transmission - Second Update.

[24] Hippich M, Holthaus L, Assfalg R, Zapardiel-Gonzalo J, Kapfelsperger H, Heigermoser M, Haupt F, Ewald DA, Welzhofer TC, Marcus BA, Heck S, Koelln A, Stock J, Voss F, Secchi M, Piemonti L, de la Rosa K, Protzer U, Boehmer M, Achenbach P, Lampasona V, Bonifacio E, Ziegler A (2021). A public health antibody screening indicates a 6-fold higher SARS-CoV-2 exposure rate than reported cases in children. Med (N Y).

[25] Stringhini S, Wisniak A, Piumatti G, Azman AS, Lauer SA, Baysson H, De Ridder D, Petrovic D, Schrempft S, Marcus K, Yerly S, Arm Vernez I, Keiser O, Hurst S, Posfay-Barbe KM, Trono D, Pittet D, Gétaz L, Chappuis F, Eckerle I, Vuilleumier N, Meyer B, Flahault A, Kaiser L, Guessous I (2020). Seroprevalence of anti-SARS-CoV-2 IgG antibodies in Geneva, Switzerland (SEROCoV-POP): A population-based study. Lancet.

