# Supplementary material for: Prevalence of SARS-CoV-2 Infection in Children by Antibody Detection in Saliva: Protocol for a Prospective Longitudinal Study (Coro-Buddy)
Source: JMIR Res Protoc. 2021 Oct 8;10(10):e27739. doi: 10.2196/27739 (PMC8510152; doi:10.2196/27739)
Supplement: Multimedia Appendix 2 [file resprot_v10i10e27739_app2.pdf]

EBERHARD KARLS UNIVERSITÄT TÜBINGEN

Universitätsklinikum Tübingen

FORMULAR 5:

Fragebogen zu:

Coro-Buddy – Längsschnittstudie zur Prävalenz von Antikörpern gegen SARS-CoV-2 in Tübinger Kindern

Studienteilnehmer ID: ....
(WIRD VOM STUDIENTEAM AUSGEFÜLLT)

BITTE ALLE GRAUEN FELDER AUSFÜLLEN!

Vor- und Nachname des Kindes/Studienteilnehmers:	....
Geburtsdatum:	....
Name der Einrichtung:	....
Datum der Befragung:	....

Sehr geehrte Eltern, sehr geehrte Erziehungsberechtigte, sehr geehrte Studienteilnehmer,

unsere Erhebungen zur Abschätzung des Anteils der Kinder, die schon mit SARS-COV-2 infiziert waren, möchten wir gerne mit 6 Fragen bzw. Informationen zu potentiellen SARS-COV-2 Kontakten innerhalb eines Haushalts sowie zu Anzeichen möglicher durchlittener Infektionen bzw. Erkältungen ergänzen. Letzteres dient uns auch dazu, den Anteil potentieller falsch positiver Antikörper, verursacht durch Erkältungsviren, abzuschätzen.

Hierzu bitten wir Sie den Fragebogen anbei vollständig auszufüllen. Zur zeitlichen Aktualisierung der Informationen werden wir Ihnen diesen Fragenbogen auch nochmal zum zweiten und dritten Speichelsammeltermin zukommen lassen - mit der Bitte diesen dann erneut auszufüllen.

Alle Daten werden pseudonymisiert abgespeichert und vertraulich behandelt. Sehen Sie bitte auch die Informationen zum Umgang mit in einer Studie erhobenen Daten im Informationsformular.

Herzlichen Dank für Ihre Teilnahme und Ihre Unterstützung!

WICHTIG: **Die folgenden Fragen beziehen sich auf den Haushalt, in dem das Kind/der Studienteilnehmer überwiegend wohnt.**

EBERHARD KARLS UNIVERSITÄT TÜBINGEN

Universitätsklinikum Tübingen

Studienteilnehmer ID:	…………	Datum der Befragung:	__.__./__ __ / __ __ __ __

1. **Wie viele Personen (inkl. Ihrem Kind/Studienteilnehmer) leben in Ihrem Haushalt ?**

   BITTE ANKREUZEN: 2: ☐ oder 3: ☐ oder 4: ☐ oder 5: ☐ oder 6: ☐ oder __: ☐

2. **Wie viele Personen sind in diesem Haushalt**

   a) erwachsen (≥18 Jahre alt): ___ *BITTE ANZAHL ANGEBEN*
   b) minderjährig (< 18 Jahre alt): ___ *BITTE ANZAHL ANGEBEN*

3. **Wurde <u>Ihr Kind/der Studienteilnehmer</u> in den letzten 6 Monaten <u>positiv auf Corona</u> (auch als SARS-COV-2 oder COVID-19 bezeichnet) getestet?**

   a) *BITTE ANKREUZEN:* Ja: ☐ oder Nein: ☐
   b) Falls JA: Können Sie bitte das (ungefähre) Datum angeben? __.__./__ __ / __ __ __ __

4. **Zeigte <u>Ihr Kind/der Studienteilnehmer</u> in den letzten 2 Monaten erkältungsartige Symptome?**

   a) *BITTE ANKREUZEN:* Ja: ☐ oder Nein: ☐
   b) Falls JA: *BITTE DAS (UNGEFÄHRE) DATUM ANGEBEN*: __.__./__ __ / __ __ __ __
   c) Falls JA: *BITTE ZUTREFFENDES ANKREUZEN:* Fieber (> 37,5°C):☐
   Halsschmerzen: ☐
   Husten: ☐
   Schnupfen: ☐
   Sonstiges ☐:____________________________

5. **Wurde in den letzten 6 Monaten <u>sonst jemand aus</u> Ihrem Haushalt <u>positiv auf Corona</u> (auch als SARS-COV-2 oder COVID-19 bezeichnet) getestet?**

   a) *BITTE ANKREUZEN:* Ja: ☐ oder Nein: ☐
   b) Falls JA: Erwachsene (≥18 Jahre alt): ___ *BITTE ANZAHL ANGEBEN*
   Minderjährige (< 18 Jahre alt): ___ *BITTE ANZAHL ANGEBEN*
   c) Können Sie bitte das (ungefähre) Datum angeben? __.__./__ __ / __ __ __ __

6. **Hatte jemand in den letzten 6 Monaten aus Ihrem Haushalt starken Geschmacks und/oder Riechverlust?**

   a) *BITTE ANKREUZEN:* Ja: ☐ oder Nein: ☐
   b) Falls JA: Erwachsene (≥18 Jahre alt): ___ *BITTE ANZAHL ANGEBEN*
   Minderjährige (< 18 Jahre alt): ___ *BITTE ANZAHL ANGEBEN*
   c) Können Sie bitte das (ungefähre) Datum angeben? __.__./__ __ / __ __ __ __
